# A Study on the Effects of Calcium Lactate on the Gelling Properties of Large Yellow Croaker (*Pseudosciaena crocea*) Surimi by Low-Field Nuclear Magnetic Resonance and Raman Spectroscopy

**DOI:** 10.3390/foods11203197

**Published:** 2022-10-13

**Authors:** Shangyuan Sang, Xiaoyun Chen, Ying Qin, Li Tong, Changrong Ou

**Affiliations:** 1 College of Food and Pharmaceutical Sciences, Ningbo University, Ningbo 315832, China; 2 Key Laboratory of Animal Protein Food Deep Processing Technology of Zhejiang Province, Ningbo University, Ningbo 315832, China

**Keywords:** large yellow croaker, calcium lactate, surimi, low-field nuclear magnetic resonance, Raman spectroscopy

## Abstract

Divalent calcium ions (Ca^2+^) are often used in surimi gels to improve their physicochemical characteristics. The present study aimed to investigate the effect of calcium lactate on the physicochemical properties, state distribution of water, and protein structure changes of surimi gels made from large yellow croaker. The results showed that the addition of calcium lactate (0%, 0.5%, 1.5%, 2.5%, 3.5%, and 4.5% on wet surimi) significantly (*p* < 0.05) increased gel strength and whiteness, while cooking loss decreased. The water-holding capacity increased first and then decreased. When calcium lactate was added to 1.5%, the water-holding capacity reached the best value. Using low-field nuclear magnetic resonance to study the distribution of water state, the bound water content first increased and then decreased with the addition of calcium lactate, reaching the highest at 1.5%. In addition, the relaxation time of immobilized water was shortest at the addition of 1.5% calcium lactate. Analyzing the protein structural changes by Raman spectroscopy showed that there was a significant decrease (*p* < 0.05) in the α-helix accompanied by an increase in β-sheets, turns, and random coils after the addition of calcium lactate. The above changes were due to the Ca^2+^ that was bound to the negatively charged myofibrils to form a protein-Ca^2+^-protein cross-linking. Therefore, the addition of calcium lactate had a significant positive effect on the gelling ability of surimi.

## 1. Introduction

Large yellow croaker (*Pseudosciaena crocea*) that is mainly limited to the coastal waters of continental East Asia is one of the three top commercial marine fishes of China [1]. It is rich in protein and fat as well as essential amino acids and polyunsaturated fatty acids. Large yellow croaker resources are very abundant in China [2]. In 2020, the seawater aquaculture production of the large yellow croakers had reached 21,353,100 tons in China. Due to the sales and transportation in the form of living life, and due to the external temperature and transportation time restrictions, its freshness cannot be effectively guaranteed. Therefore, the deep processing of the large yellow croaker into surimi can solve the problem of its decay in a short time and enrich its product form. The process of surimi involves taking meat, chopping, washing, dehydration, refining, as well as bagging. The most common types of surimi products in China’s freshwater surimi market are fish balls, fish cake, fish rolls, fish intestines, etc. These products can be transported and stored in the low hand environment of minus 20 °C, suitable for urban family consumption, convenient production, and fresh and tender taste. In 2020, the production of surimi product was 1,267,727 tons in China [3]. It has a significant market consumption potential. However, the high fat content of large yellow croakers would be averse to surimi gelatin. Polyphosphates are generally used to improve the quality of surimi gels [4,5], while high doses of them can cause kidney damage and interfere with the absorption of calcium [6].

The characteristics of the physics of surimi gels is one of the important aspects of studying its quality, which is closely related to the function of myofibrillin, and further changes its microstructure. Therefore, calcium ions (Ca^2+^) were added to improve the gelling properties of croaker surimi in this study as Ca^2+^ can increase the protein–protein interactions via the formation of a salt-bridge between negatively charged myofibrillar proteins [7,8]. In addition, different forms of calcium salts, such as lactate, citrate, sulfate, and caseinate, have been known to activate indigenous transglutaminase, which can catalyze the cross-linking reaction between γ-carboxyamide groups of glutamine and ε-amino groups of lysine to form a ε-(γ-glutamyl) lysine linkage, leading to the formation of stronger gel [8]. Calcium lactate, an excellent additive naturally present in the human body, has a higher solubility and bioavailability than calcium gluconate, calcium citrate, calcium carbonate, and calcium phosphate [9]. Previous studies have shown that calcium lactate can improve the textural properties of fish samples [10]. In addition, as one of the potential natural sources of antibacterial agents, the product of lactic acid itself has an antibacterial effect [11]. However, there is little information about the effect of calcium lactate on the characteristics of surimi of large yellow croaker.

Low-field nuclear magnetic resonance (LF-NMR) can be used to qualitatively and quantitatively analyze the water distribution and water mobility in food according to the spin–spin relaxation time (T2) of samples [12]. Raman spectroscopy can be used for rapid nondestructive detection of protein structure and qualitative and quantitative analysis of changes in protein functional groups [13]. Therefore, in this paper, LF-NMR and Raman spectroscopy were used to study the effect of calcium lactate on the protein structure and water status of large yellow croaker, as well as the relationship between calcium lactate and surimi gel characteristics parameters, to provide a theoretical basis for the quality evaluation and improvement of surimi products.

## 2. Materials and Methods

### 2.1. Materials

Dozens of 500 g large yellow croakers were purchased from local aquatic markets (Ningbo, China) and brought to the laboratory covered with ice cubes within 24 h. Calcium lactate is food grade and offered by Jindan Lactic Acid Technology Company (Zhoukou, China).

### 2.2. Cooking Loss

Cooking loss (CL) was measured using a method described by Yang, Wang, Wang, and Ye [14]. Before and after heating (90 °C, 20 min), the weight of surimi gel was G1 and G2, respectively. The CL was represented as the loss of liquid:

CL (%) = (G1 − G2)/G1 × 100
(1)

### 2.3. Preparation of Surimi Gels

Fish head, skin, dark muscle, and bones were removed manually and the flesh was minced to uniformity using a mincer within about 2 min. The mince was washed at a water/mince ratio of 4:1 (*w*/*w*) and then stirred for 10 min. The homogenate was centrifuged at 4000× *g* for 10 min at 4 °C after washing. Washed mince was chopped for 10 min and chopped with 2% NaCl (*w*/*w*, on fish mince) for another 10 min at 4 °C. Then, the chopped mince continued to be chopped for 5 min with starch (3%, *w*/*w*, on fish mince) and calcium lactate (0%, 0.5%, 1.5%, 2.5%, 3.5%, and 4.5%, *w*/*w*, on fish mince). Finally, all the surimi samples were stuffed into a casing and heated immediately for 1 h at 40 °C and then for 30 min at 90 °C in a water bath.

### 2.4. Color Evaluation

Three gel samples of each treatment were subjected to color evaluation using a HunterLab (CM-400d, Konica Minolta, Tokyo, Japan). Illuminant C was used as the light source of every measurement. Measurement of L* (lightness), a* (redness/greenness), and b* (yellowness/blueness) values was conducted. Whiteness was calculated using Equation (2) [15]:

Whiteness = 100 − [(100 − L*)^2^ + (a*)^2^ + (b*)^2^]^1/2^ (2)

### 2.5. Gel Strength

Gel strength was measured according to the method of Abe, Asada, and Kajiwara using a texture analyzer (Model TA-XT2, Stable Micro Systems, Godalming, Surrey, UK) [16]. Gels were cut into cylinder-shaped samples with a diameter of 25 ± 1 mm and height of 15 ± 1 mm and equilibrated at room temperature (25 °C). A probe model of the P/0.5 s spherical probe, a maximum displacement of 15 mm, a trigger force of 5.0 g, and a test speed of 1 mm/s were set as the main parameters. Each type of sample was determined in triplicate. Gel strength is also known as elasticity as a significant indicator that represents the gel forming ability of surimi. The gel strength was calculated according to Equation (3). The value of the breaking force was read at the first peak force (g) according to the force–time deformation curve. The breaking distance was the distance traveled by the probe from the surface of the sample to the point of breakage.

Gel strength (g × cm) = breaking force (g) × breaking distance (cm)
(3)

### 2.6. Water-Holding Capacity of Surimi Gels

The water-holding capacity (WHC) was measured using a method described by Sánchez-González with slight modifications [17]. A 1.5 g sample of surimi packed with a piece of filter paper was introduced into centrifuge tubes and centrifuged at 4900× *g* (TDL 50C, Anting Scientific Instrument Factory, Shanghai, China) for 15 min at room temperature. The mass m1 (g) before centrifugation and the mass m2 (g) after centrifugation were measured, and the WHC was m2/m1 × 100%. Measurements were determined in triplicate.

### 2.7. LF-NMR Measurement of Surimi Gels

The LF-NMR relaxation measurements were performed on a Niumag Micro MR 20-025 (Niumag Electric Corporation, Suzhou, China) operating at a resonance frequency for hydrogen protons of 18.17 MHz. An approximately 5.6 g sample was placed in a 25 mm glass tube and inserted in the LF-NMR probe. A Carr–Purcell–Meiboom–Gill (CPMG) pulse sequence was employed to measure the spin–spin relaxation time, and the pulse parameters were as follows: P90 (µs) = 5.50, P180 (µs) = 11.00, SW (kHz) = 200, D3 (µs) = 20, TR (ms) = 2000, RG1 = 20, RG2 = 3, NS = 4, EchoTime (µs) = 250, and EchoCount = 10,000.

### 2.8. Raman Spectroscopy of Surimi Gels

The Raman spectra of surimi gels were recorded by a spectrometer equipped with a 50× lens as a microscope (Ningbo Institute of Industrial Technology, Ningbo, China). It was used to focus the excitation laser beam (532 nm exciting line of a Spectra Physics Ar-laser) on the sample spread on a glass slide and collected the Raman signal in the backscattered direction. The laser power was controlled at about 12 mW and the laser spot diameter was about 1 µm at the sample surface. The Raman spectra were recorded in the range of 200–3650 cm^−1^. Each spectrum according to 30 scans, 20 s exposure time, and 1 cm^−1^ resolution conditions was obtained. Furthermore, Raman spectra were baseline-corrected and normalized against the phenylalanine band at 1003 cm^−1^ using the LabSpec Application.

Raman spectroscopic data were then analyzed with the PeakFit v4.12. Fourier reflexive convolution, second-derivative analysis, and band curve fitting are three methods to open and sharpen overlapping hidden peaks. In this experiment, PeakFit V4.12 was used to perform nonlinear fitting of peaks in spectral data to quantitatively calculate the area of each secondary structure, and then the second-derivative spectrum was used to locate overlapping hidden peaks. Finally, the curve fitting function was used to calculate the area of each peak, reported as a percentage [12].

### 2.9. Statistical Analysis

All the results were in triplicate and statistically analyzed by the SPSS 8.0 software. (International Business Machines Corporation, Chicago, IL, USA). Analysis of variance (ANOVA) was employed to determine the significance of main effects. Significant differences (*p* < 0.05) between means were identified using Duncan’s multiple range test. Correlations between different indices were analyzed using Pearson type.

## 3. Results and Discussion

### 3.1. CL of Surimi

The CL of large yellow croaker surimi gels with added calcium lactate (0%, 1.5%, 2.5%, 3.5%, and 4.5%, *w*/*w*) is shown in Table 1. The CL of the control sample was higher than the sample with a calcium lactate content over 3.5% and lower than the sample with a calcium lactate content less than 2.5% (*p* < 0.05). This suggested that adding calcium lactate surpassing 3.5% could prevent the water loss during cooking and that it had a good protective effect on the CL stability compared with the control sample.

### 3.2. Whiteness of Surimi Gels

The effects of calcium lactate on whiteness, L* (lightness), a* (redness/greenness), and b* (yellowness/blueness) of large yellow croaker surimi gels are shown in Table 1. The different calcium lactate contents resulted in the change in color properties of surimi gels. The whiteness of the surimi gels added with 0.5–4.5% calcium lactate was 75.07–77.58, all higher compared with the control gels of 74.87 (*p* < 0.05). Whiteness is an important parameter to determine the quality of surimi, with values greater than 75 generally considered acceptable [18,19]. In addition, with increasing calcium lactate contents in surimi, the results showed rising values of whiteness. Therefore, adding calcium lactate might improve the whiteness of surimi gels.

### 3.3. Strength of Surimi Gels

**Figure 1:**
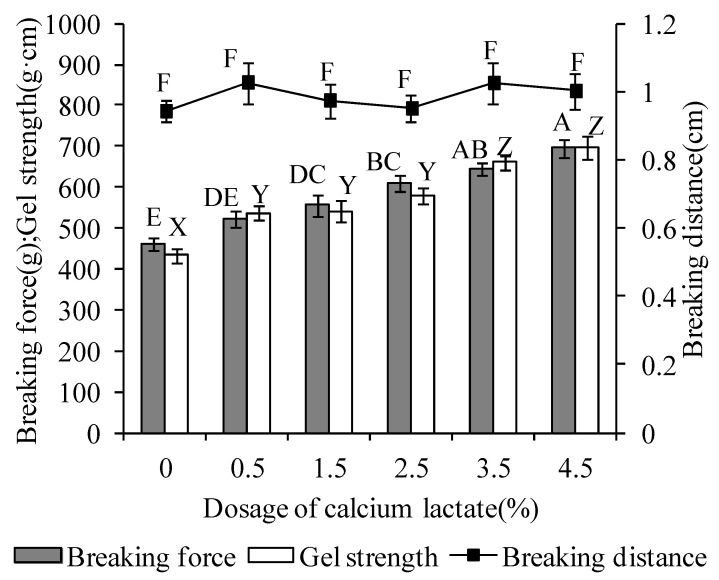
Effect of calcium lactate on breaking force, breaking distance, and gel strength. A–E: different letters at breaking force differ significantly (*p* < 0.05). X–Z: different letters at gel strength differ significantly (*p* < 0.05). F: indicates no significant difference (*p* > 0.05) at breaking distance.

The effect of calcium lactate on the gel strength of surimi of large yellow croaker is shown in Figure 1. Compared with the control group, the addition of calcium lactate significantly enhanced the gel strength in the range of 0.5–4.5% and reached the maximum gel strength at 4.5% (*p* < 0.05). These results are consistent with the report of Lee and Park (1998) [9]. Calcium lactate not only has good water solubility, but divalent calcium ions may also strengthen the fixation of the tissue structure, acting equally with monovalent salt ions on surimi gels. Thus, calcium lactate obviously affected the gel strength of surimi to improve its quality [19].

### 3.4. WHC of Surimi Gels

WHC is an important parameter as the capacity of surimi gels to retain water in the microstructure. In Table 1, there is a significant difference (*p* < 0.05) in WHC between calcium-lactate-added groups and the control. The surimi gels with low addition levels (0.5–1.0%) of calcium lactate has a higher WHC than the control gels. By contrast, high addition levels (1.5–4.5%) of calcium lactate reduce the WHC of surimi gels. The WHC of surimi improved by a certain concentration of Ca^2+^ at the appropriate temperature, which could activate transglutaminase in surimi and catalyze the cross-linking between the carboxyl amide groups in glutamic acid residues and other amino acid residues [20].

### 3.5. Water Distribution of Surimi Gels

LF-NMR as a rapid, nondestructive monitoring method was used to investigate changes in water mobility during food processing by measuring proton relaxation [21,22]. The proton spin–spin relaxation time (T2) indicates various statuses of water and is directly related to the mobility of water in food [11]. The properties of water states and the distribution of large yellow croaker surimi gels were evaluated by the LF-NMR.

**Figure 2:**
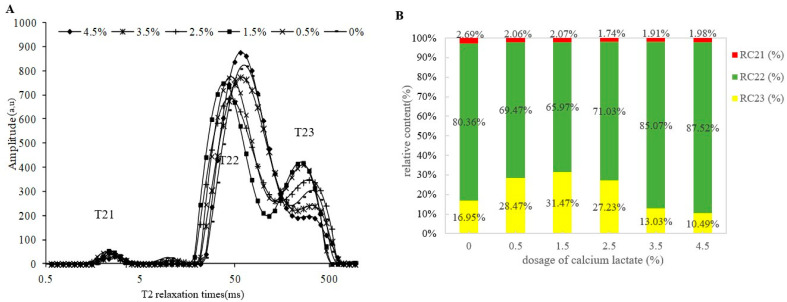
Effect of different dosages of calcium lactate on T2 relaxation time (**A**) and the relative content (**B**) of bound water (RC21), immobilized water (RC22), and free water (RC23) of large yellow croaker surimi gels.

Figure 2A shows the effect of calcium lactate on the T2 distribution of large yellow croaker surimi gels. A range (T22) with a relaxation time around 30–100 ms is known as the immobilized water and a small range at about 200–400 ms (T23) is free water. The range at 1–15 ms (T21) was on behalf of the bound water with large molecules such as proteins in the gel system [15]. In Table S1, the relaxation time peaks of T21, T22, and T23 populations significantly decreased in most of the calcium lactate-added surimi (*p* < 0.05) compared with the control group, indicating that water mobility also diminished in the surimi after adding calcium lactate. The results also indicated that the addition of calcium lactate had a significant effect on the T23 (*p* < 0.05) but had no significant effect on the T21 (*p* > 0.05). When the addition level of calcium lactate was less than 1.5%, the T23 of surimi gels was shortened. Thus, a proper amount of calcium lactate can limit the flowing of water in the surimi.

Comparison of the continuous distribution profiles revealed clear differences in the distribution of water mobility between the addition of calcium lactate and control groups. The relative content of immobilized water (RC22) decreased first to the lowest at 1.5% calcium lactate and then increased with the addition of 2.5–4.5% calcium lactate (Table S1). According to the experimental results, the free water content (RC23) of surimi gels with 0.5–2.5% calcium lactate was higher than the control group, while the 3.5–4.5% calcium lactate group was lower than the control group.

### 3.6. Raman Spectroscopy of Proteins in Surimi Gels

Raman scattering spectroscopy was applied in this study to explain the backbone conformations of protein molecules in surimi gels. Changes in the Raman bands of protein chemical groups provided the information of changes in the secondary and tertiary structure of proteins [23,24,25].

**Figure 3:**
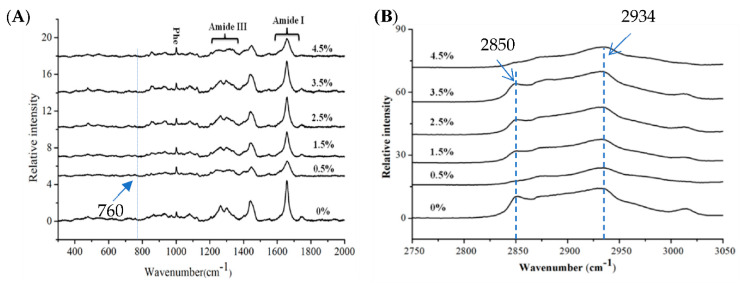
Original Raman spectrum (300–2000 cm^−^^1^ (**A**) and 2750–3050 cm^−^^1^ (**B**)) of large yellow croaker surimi gels with various dosages of calcium lactate.

In this present study, a detailed spectral analysis provided a valuable tool for the study of large yellow croaker surimi gels with calcium lactate addition. Raman spectra of large yellow croaker surimi gels with various concentrations of calcium lactate (0%, 1.5%, 2.5%, 3.5%, and 4.5% *w*/*w*) in the region are shown in Figure 3. The assignments of the corresponding bands are included in Table S2 [24,26,27].

#### 3.6.1. Changes in the Secondary Structure of Protein

The Raman band that provides information about the secondary structure of surimi gels is amide III, which mainly involves C-N stretching, N-H in-plane bending, C_α_-C stretching, and C=O in-plane bending vibrations of the peptide bond [27]. It was difficult to interpret the amide III band as the vibrational spectroscopy of surimi gel protein produced a complex pattern of bands in the 1225–1350 cm^−1^ region (Figure 3A, Table S2). The intensity of the α-helix structure representing this band around 1260–1300 cm^−1^ overlapped with the range of turns. Despite the β-sheet and random coil bands superposition in the amide III region (1250 and 1240 cm^−^^1^), an increasing intensity can be observed in the range of 1225–1240 cm^−^^1^ in calcium-lactate-added gels. These alterations in the secondary structure content and structural properties of the proteins can be attributed to the β-sheet formed by calcium lactate addition, because it forms high-molecular-weight complexes with a compact and ordered conformation.

**Figure 4:**
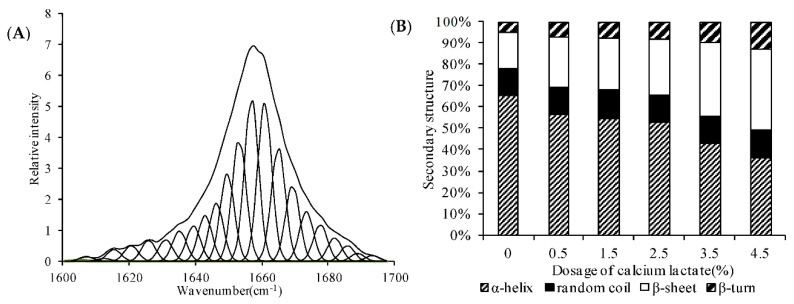
Original Raman spectrum (above) at the amide I band and its corresponding deconvolved spectrum (below) fitted with Gaussian components (**A**). The estimated secondary structure fractions (**B**) of proteins in large yellow croaker surimi gels with various dosages of calcium lactate.

The Raman band around 1657 cm^−^^1^ was part of the amide I (1600–1700 cm^−^^1^) vibrational mode (Table S2, Figure 4A), which directly provides secondary structural information about protein and involves mainly C=O stretching, C_α_-C-N bending, C-N stretching, and N-H in-plane bending of peptide groups [23,24,25,28]. In this study, the highest intensity was at the 1657 cm^−^^1^ band in the spectra of large yellow croaker surimi gels with various calcium lactate concentrations. That can be put down to the high α-helix content (Figure 4A). A rational excuse for the band change may be the reduction in α-helix content resulting from calcium lactate addition.

Quantitative analysis of the protein secondary structure was performed by using Fourier reflexive convolution, second-derivative analysis, and band curve fitting. After spectral normalization, the intensity values of Raman bands from various atomic groups were determined. The visible bands were assigned to vibrational modes of amino acid side chains or peptide backbones carried out by comparing Raman spectra of model polypeptides or monographs of Raman spectra of proteins [27]. Figure 4A shows the original and deconvolved spectrum of large yellow croaker surimi gels. Then, the secondary structure fractions of the surimi gels were estimated, respectively, on the basis of the area of appropriate peaks. Similar results were also discovered in heat-induced gelation [27,29,30]. The spectral profiles and percentage of the secondary structure determined from the amide I spectral profile for large yellow croaker surimi gels with different contents of calcium lactate are shown in Figure 4B. There were remarkable (*p* < 0.05) differences in the α-helix, β-sheets, and turns structure contents after calcium lactate was added. There was a significant decrease (*p* < 0.05) in the α-helix accompanied by increased β-sheets, turns, and random coils. Upon 4.5% calcium lactate addition, the content of α-helix decreased from 65.82% to 36.19%, and β-sheets, turns, and random coils increased from 17.13%, 4.87%, and 12.18% to 37.86%, 12.97%, and 12.98% (*p* < 0.05), respectively. Higher contents of β-sheets and turns in the presence of calcium lactate indicated that the secondary structure of large yellow croaker surimi gels was likely to be calcium-lactate-dependent.

#### 3.6.2. Local Environments of Protein Networks in the Surimi Gels

There are several Raman bands that have characteristics of the tertiary structure of surimi gels. Changes in these bands could monitor the polarity of the local environment or involvement in hydrogen bonding [26]. In this study, the normalized intensity of the Raman band near 760 cm^−^^1^ in the large yellow croaker surimi gels with 0.5% calcium lactate (0.20) showed a significant decrease (*p* < 0.05) compared with the control (0.36) (Figure 3A), which indicated that the hydrophobicity was involved in the surimi gels and the hydrophobic microenvironment became exposed to the polar aqueous solvent with calcium lactate addition.

The doublet bands located near 830 and 850 cm^−^^1^ could monitor the microenvironment around tyrosine residues and be assigned to vibrations of the para-substituted benzene ring of tyrosine residues, which were affected by the environment and the involvement of the phenolic hydroxyl group in hydrogen bonding [31]. I850/I830 is the doublet bands intensity ratio that has been proposed to determine whether the tyrosine residue is solvent-exposed or buried [28]. The ratio increased from 1.27 to 2.09 as the addition level of calcium lactate increased from 0 to 4.5%, which suggested that the tyrosine residues were exposed to the aqueous or polar microenvironment or acted as a simultaneous acceptor and donor of moderate to weak hydrogen bonds.

Proteins exhibited C-H stretching vibration (νC-H) in the 2800–3050 cm^−^^1^ band of the Raman spectrum [32]. There were two peaks at 2850 cm^−^^1^ and 2934 cm^−1^ in the νC-H band of the control sample, while one peak at 2850 cm^−1^ of the sample with 0.5% calcium lactate vanished (Figure 3B). During gelation with the addition of calcium lactate, the unfolding of the protein may result in the exposure of more methyl or methylene groups. With the increase in calcium lactate level, the strength and area of the νC-H band decreased first and then increased. These changes suggested an increased hydrophobic interaction of surimi gels with 0.5% calcium lactate, and protein cross-linking may be due to such hydrophobic contacts between protein side-chains.

### 3.7. Correlations Analysis

The Pearson correlation coefficient can determine whether there is a linear relationship between the two indicators. Table 2 shows the correlation between the physical properties of gels and the secondary structure of surimi protein, and it was found that both were related. The results showed that WHC was mainly negatively and significantly correlated with T23 (correlation coefficient −0.68), the peak area fraction of RC22 (correlation coefficient −0.75), and β-sheet and β-turn content (correlation coefficients −0.88 and −0.84, respectively). It was positively and significantly correlated with the peak area fraction of RC23 (correlation coefficient 0.73), RC21 (correlation coefficient 0.52), α-helix content (correlation coefficient 0.85), and CL (correlation coefficient 0.78), with a highly significant positive correlation with α-helix content and CL. GS was positively correlated with whiteness (correlation coefficient 0.94), RC22 (correlation coefficient 0.51), and β-sheet and β-turn content (correlation coefficients 0.99 and 0.96, respectively) and negatively correlated with WHC (correlation coefficient −0.87), RC21 (correlation coefficient −0.74), and α-helix (correlation coefficient −0.99), with a highly significant negative correlation with α-helix. Stangierski et al. also pointed out that the variation in the qualitative parameters was determined by the relative content of free and bound water, which can be confirmed by the relaxation time values [33]. Thus, it was seen that there was a strong correlation between structure and function, and WHC was strongly correlated with LF-NMR relaxation properties and protein secondary structure.

## 4. Conclusions

Calcium lactate had significant effects on the quality of large yellow croaker surimi gels, including gel strength, water-holding capacity, and cooking loss. LF-NMR and Raman spectroscopy had also been proven useful tools for the study of the structures of large yellow croaker surimi gels fortified with calcium lactate. In our study, we thoroughly investigated changes in the relaxation time, content of bound water, immobilized water, and free water as well as the reduction in α-helix and increase in β-sheets, β-turns, and random coils. In addition, significant correlations were found between structural changes in surimi gel proteins and some other indices of surimi gels using the Pearson type of SPSS system. These results provided evidence that the addition of calcium lactate had a significant influence on the property of large yellow croaker surimi gels, and also suggested that the formation of protein-calcium-protein might occur.

Furthermore, due to the experimental cycle and objective conditions, there are still many deficiencies and imperfections in this paper. It is suggested that the following work can be carried out in the future: (1) study the effect of calcium lactate on protein aggregates (or tertiary structure) and their molecular weight distribution; (2) contrast-study the impact of anions or other cations (such as zinc and iron) on surimi gel products and their impact on human health.

## Figures and Tables

**Table 1:** Effect of calcium lactate on cooking loss, water-holding capacity, whiteness, L*, a*, and b*.

Calcium Lactate (%)	Cooking Loss (%)	Water-Holding Capacity (%)	Whiteness	L*	a*	b*
0	12.13 ± 0.007 c	77.86 ± 0.01 bc	74.87 ± 0.64 a	75.31 ± 0.62 a	−2.75 ± 0.09 d	3.71 ± 0.54 d
0.5	19.85 ± 0.003 e	78.72 ± 0.03 c	75.07 ± 0.43 b	75.47 ± 0.41 a	−2.55 ± 0.13 e	3.67 ± 0.39 d
1.5	20.17 ± 0.010 e	78.89 ± 0.01 c	75.92 ± 0.63 b	76.43 ± 0.66 ab	−2.67 ± 0.12 c	3.86 ± 0.46 e
2.5	16.67 ± 0.005 d	74.01 ± 0.04 b	76.84 ± 0.56 c	77.21 ± 0.55 c	−2.92 ± 0.13 b	2.82 ± 0.49 c
3.5	10.02 ± 0.003 b	71.68 ± 0.04 a	77.48 ± 0.54 d	77.91 ± 0.56 d	−3.06 ± 0.10 a	1.86 ± 0.13 a
4.5	8.47 ± 0.002 a	71.12 ± 0.02 a	77.58 ± 0.36 d	77.71 ± 0.35 d	−2.95 ± 0.15 b	1.97 ± 0.37 bc

Results are presented as the mean ± standard deviation. Different letters (a–e) in the same column represent significant differences (*p* < 0.05). L*, lightness; a*, redness/greenness; b*, yellowness/blueness.

**Table 2:** Pearson correlations among different indices of large yellow croaker surimi gels.

	T	WHC	CL	GS	Whiteness	RC21	RC22	RC23	T21	T22	T23	α-Helix	Coil	β-Sheet	β-Turn
**T**	1	−0.9342	−0.6099	0.9661	0.9809	−0.6562	0.5863	−0.5630				−0.9644		0.9637	0.9533
**WHC**	−−	1	0.7836	−0.8720	−0.9397	0.5192	−0.7559	0.7325			−0.6870	0.8558		−0.8831	−0.8408
**CL**	−	++	1		−0.5739		−0.9883	0.9877		−0.8512	−0.9535	0.5410		−0.5770	−0.5432
**GS**	++	−−		1	0.9376	−0.7355	0.5088		−0.5886			−0.9896		0.9926	0.9643
**Whiteness**	++	−−	−	++	1	−0.705	0.5357	−0.5104	−0.5007			−0.9128		0.9212	0.8819
**RC21**	−	+		−	−	1			0.9114	0.5796		0.6519		−0.6492	−0.6200
**RC22**	+	−−	−−	+	+		1	−0.9991		0.8543	0.9335	−0.5550		0.5943	0.5529
**RC23**	−	+	++		−		−−	1		−0.8685	−0.9327	0.5324		−0.5705	−0.5308
**T21**				−	−	++			1	0.6230		0.5213		−0.5047	−0.5506
**T22**			−−			+	++	−−	+	1	0.9149		−0.5993		
**T23**		−	−−				++	−−		++	1		−0.6059		
**α** **-helix**	−−	++	+	−−	−−	+	−	+	+			1		−0.9956	−0.9859
**Coil**										−	−		1		
**β** **-sheet**	++	−−	−	++	++	−	+	−	−			−−		1	0.9730
**β** **-turn**	++	−−	−	++	++	−	+	−	−			−−		++	1

T, the treatment, at different addition levels of calcium lactate in the surimi gels; WHC, water-holding capacity; CL, cooking loss; GS, gel strength; Coil, random coil; RC21, RC22, RC23, the relative contents of bound water, immobilized water, and free water in the surimi gels, respectively; T21, T22, T23, the lateral relaxation times of protons from bound water, immobilized water, and free water, respectively; positive signs (+) mean positive correlations, whereas negative signs (−) mean negative correlations. Single sign indicates a correlation between two indices (0.5 ≤ |R| < 0.75). Double signs represent a higher correlation (0.75 ≤ |R| < 1).

## Data Availability

Data are contained within the article or Supplementary Material.

## Supplementary Materials

The following supporting information can be downloaded at: https://www.mdpi.com/article/10.3390/foods11203197/s1, Table S1: Effect of calcium lactate on T2 and RC2; Table S2: Tentative assignment of some bands in the Raman spectra of surimi gels.

## Glossary

CL	Cooking loss
LF-NMR	Low-field nuclear magnetic resonance
WHC	Water-holding capacity
GS	Gel strength
