# Supplementary material for: A Study on the Effects of Calcium Lactate on the Gelling Properties of Large Yellow Croaker (Pseudosciaena crocea) Surimi by Low-Field Nuclear Magnetic Resonance and Raman Spectroscopy
Source: Foods. 2022 Oct 13;11(20):3197. doi: 10.3390/foods11203197 (PMC9602013; doi:10.3390/foods11203197)
Supplement: Supplementary file 1 [file foods-11-03197-s001.zip › foods-1924936-supplementary1.pdf]

Table S1. Effect of calcium lactate on T2 and RC2.

Calcium lactate (%)	T21 (ms)	T22 (ms)	T23 (ms)	RC21 (%)	RC22 (%)	RC23 (%)
0.00	2.67±0.16 c	65.79±4.04 d	351. 12±0.00 d	2.69±0.03 e	80.36±0.39 d	16.95±0.41 c
0.50	2.33±0.26 a	43.29±0.00 b	265.61±0.00 b	2.06±0.06 d	69.47±0.46 b	28.47±0.46 d
1.50	2.44±0.30 b	37.65±0.00 a	242.54±16.31 a	2.77±0.17 d	65.27±0.21 a	31.47±0.10 e
2.50	2.23±0.30 a	43.29±0.00 b	305.39±0.00 c	1.74±0.09 a	71.03±0.27 c	27.23±0.25 d
3.50	2.44±0.30 b	57.22±4.04 c	351.12±0.00 d	1.91±0.20 b	85.07±0.38 e	13.03±0.20 b
4.50	2.33±0.22 a	57.22±0.00 c	351. 12±0.00 d	1.98±0.21 c	87.52±0.96 f	10.49±1.21 a

Results are presented as the mean ± standard deviation. T21, T22, T23 mean the lateral relaxation time of bound water, immobilized water, free water; RC21, RC22, RC23 mean the relative content of bound water, immobilized water, free water. Different letters (a-f) in the same column represent significant differences ($P < 0.05$).

Table S2. Tentative assignment of some bands in the Raman spectra of surimi gels.

Frequency (cm-1)	Assignment
I825/852	Tyr doublet stretching ring
758	Trp stretching ring
882	
932	νC-C (α-helix)
1003	Phe stretching ring
1034	Phe stretching ring
1063	CN or CH stretching
1126	CN stretching
1208	Tyr or Phe
1244 (Amide III)	β-sheet
1304 (Amide III)	α-helix
1322	CH deformation, Trp
1340	
1410	COO-, Asp, Glu, CH deformation stretching
1453	CH3, CH2, CH bending
1600– 1700 (Amide I)	1655±5 α-helix<br>1670±5 antiparallel β-sheet<br>1665±5 random coil<br>1685 β-turn
2936	CH stretching amino acids (aromatic, aliphatic, charged)

Tyr: tyrosine; Trp: tryptophan; Phe: phenylalanine; Asp: aspartic acid; Glu: glutamic acid.
